# A Longitudinal Analysis of Mothers’ Parenting Stress and Internalizing and Externalizing Behavior of Young Children on the Autism Spectrum

**DOI:** 10.1007/s10803-024-06362-x

**Published:** 2024-05-08

**Authors:** Jessica Paynter, Vanessa Heng, Madonna Tucker, Stephanie Malone

**Affiliations:** 1 Griffith Institute for Educational Research, Griffith University, 58 Parklands Drive, Gold Coast Campus, Southport, QLD 4222 Australia; 2 School of Applied Psychology, Griffith University, Gold Coast, Australia; 3 AEIOU Foundation, Gold Coast, Australia

**Keywords:** Longitudinal, Autistic, ASD, Challenging behavior, Early intervention

## Abstract

We investigated longitudinal relations between internalizing, externalizing, and total behaviors that challenge in young children on the autism spectrum and mothers’ parenting stress. Participants included 93 mothers of children on the autism spectrum aged 27.89–65.84 months, who completed questionnaires on maternal parenting stress, and children’s internalizing (anxiety), externalizing (disruptive), and total behaviors that challenge. Data were collected on early intervention program intake and approximately one year later. Cross-sectional findings indicated small to medium effect size associations between internalizing and externalizing behavior and parenting stress. However, cross-lagged structural equation models found that neither internalizing nor externalizing behavior predicted later parenting stress, nor the reverse. Significant stability effects were found for measures of child internalizing (anxiety), externalizing (disruptive), and total behaviors, and parenting stress. Relations between behaviors that challenge and parenting stress over time were non-significant in our models that controlled for stability of behaviors and parenting stress over time. Implications for research and clinical practice, in understanding and targeting the persistence of behaviors that challenge and parenting stress, are discussed.

Parents of children on the autism spectrum^1^ report higher levels of parenting stress than other parent groups (Barroso et al., 2018; Hayes & Watson, 2013). Parenting stress is the sense of distress arising from parenting demands (Hakvoort et al., 2012). Children’s behaviors that challenge, including internalizing (e.g., anxiety) and externalizing (e.g., disruptive behaviors) behaviors, are strong, consistent, cross-sectional predictors of parenting stress (Barroso et al., 2018; Yorke et al., 2018). However, longitudinal findings are mixed (Yorke et al., 2018). Mixed longitudinal findings may be due to methodological differences across studies, or relations genuinely varying at different ages and stages of children’s lives. The purpose of the present study is to investigate the longitudinal relationships between mothers’ parenting stress and child behaviors that challenge. Our focus is on the early intervention stage when children are aged 2–5 years, which could inform supports during this key period.

Transactional models of child development predict bidrectional relationsips between parents’ stress and children’s behavior (see Rodriguez et al., 2019, for an overview). For parents, observing children’s behaviors may be distressing, leading to increased parenting stress over time. Converesly, increased stress in parents may impact how parents respond to their children, including having less resources to respond to desirable behaviors and using parenting practices that inadvertently reinforce behaviors over time. However, in a systematic review, Yorke et al. (2018) found mixed longitudinal findings into the relations between parents’ stress and children’s behavior over time.

Seven studies were included in Yorke et al.’s review (2018) that addressed bidirectional relationships longitudinally between parenting stress and child behavior. Results were mixed including positive associations in either or both directions (e.g., Zaidman-Zait et al., 2014) and null results (e.g., Peters-Scheffer et al., 2012). Studies in the review varied in controls included in analyses, measurement tools, and child ages which may account for mixed findings. For example, autism characteristics were controlled in only one paper (Osborne & Reed, 2009) with two cohorts (ages 2:6–4:0 years and 5:0–16:0 years old) over a period of 9–10 months follow-up. Osborne and Reed (2009) found child behavior was no longer a significant predictor of later parenting stress, after controlling for autism characteristics. However, parenting stress remained a significant longitudinal predictor of child behavior, after controlling for autism characteristics. This study did not control for the potential stability of parenting stress and child behavior over time. The stability of these two variables was examined in two further studies (Peters-Scheffer et al., 2012; Zaidman-Zait et al., 2014) that yielded mixed results.

Peters-Scheffer et al. (2012) found in a study with preschool children on the spectrum, null results in assessments completed every six months over two years. That is, earlier parenting stress did not predict later child behavior, nor the reverse. In contrast, Zaidman-Zait et al. (2014) found, in a sample of children on the spectrum aged 24–47 months, significant pathways for parent-to-child effects at most timepoints 12 months apart, and one significant pathway for child-to-parent effects over four time points. Taken together, at the time of Yorke et al.’s (2018) review, there were no studies that controlled for stability effects, and included potential confounds (e.g., autism characteristics) which is needed to reconcile the conflicting results. The current study addresses relations between parenting stress and children's behavior over time to address conflicting reports and expand on previous research.

Two additional longitudinal papers have been published recently that begin to address this gap, but again with mixed results. Rodriguez et al. (2019) investigated longitudinal associations across four time points over three years collected approximately 12 months apart, between child behavior (internalizing and externalizing) and parenting stress (mothers and fathers) in 188 children on the autism spectrum aged 5–12 years. They controlled for stability of each variable in analyses. Mother and father parenting stress each predicted child internalizing behavior at the next time point. In contrast, internalizing behavior did not significantly predict later parenting stress for either parent. Mixed findings for externalizing behaviors were found. In earlier timepoints parenting stress in mothers (T1, T2) significantly predicted later child externalizing behavior (T2, T3). This same pattern was also in seen in fathers for T2 to T3 (T1 to T2 was non-significant). However, externalizing behaviors (T1, T2) did not significantly predict later parenting stress in either parent (T2, T3) in these earlier time points. At the final timepoints (T3 to T4), the reverse was found for fathers only, with externalizing behaviors predicting later parenting stress, but parenting stress for either parent did not significantly predict later externalizing behavior. Thus, this study indicated that different patterns may be seen at different ages or stages of children’s development. A limitation of this study however was that while Rodriguez et al. (2019) measured autism characteristics, these were not included as controls in their models of parenting stress and child behavior.

Lin et al. (2021) is the only study to date to the authors’ knowledge that has included both stability effects and autism characteristics to investigate bidirectional longitudinal associations between child behavior and parenting stress. Participants were 75 children on the autism spectrum aged 18–42 months and their parents over two assessments 1.5 years apart in Taiwan. Measures included translated versions of the full Parent Stress Index (PSI; Weng, 2003) to evaluate parenting stress, the Child Behavior Checklist (CBCL; Achenbach & Rescorla, 2000) to measure child behavior, and the Autism Diagnostic Observation Schedule (Lord et al., 1999) module 1 total algorithm raw scores to evaluate autism characteristics. They incorporated three measures of stress using the PSI subscales: parent-related stress (e.g., competence), child-related stress (e.g., child demandingness), and total stress using the total score. Significant stability (i.e., earlier scores strongly predicted later scores) of all three measures of parenting stress and child internalizing and externalizing behaviors were found. Child behavior measures did not show significant pathways in models to later parenting stress measures. In contrast, total parenting stress and child-related stress were significant predictors of later child externalizing behavior, but not internalizing behavior. A limitation however was that the total and child-related stress measures included items about children which may overlap with measures of child externalizing behavior, therefore potentially inflating associations that were found. Evidence for inflation was observed in Yorke et al.’s (2018) review of cross-sectional studies, whereby higher correlations between total parent stress and child externalizing behavior were observed when compared to parent-specific measures of parenting stress and child externalizing behavior.

While strong cross-sectional associations between parenting stress and child behavior are well established (e.g., Yorke et al., 2018), findings for longitudinal relationships are mixed which may be due to significant methodological differences across studies and varying age groups/stages included. Lin et al. (2021) conducted the only study that controlled for stability of parenting stress and child behavior over time and autism characteristics. However, they used a child behavior measure not designed for children on the spectrum (CBCL; Achenbach & Rescorla, 2000) which may not capture the behaviors associated with autism. Furthermore, significant associations between earlier parenting stress and later child externalizing behavior may have been explained at least in part, by overlapping items in total and parent-related stress scales used for analyses that include child-related items. Therefore, in our study we sought to address these limitations. We elected to use a measure designed for children with neurodevelopmental conditions, the Developmental Behavior Checklist (DBC, Einfeld & Tonge, 2002) and to include both total scores and a more specific measure of parent-related distress analyzed separately using the PSI short form (Abidin, 1995). We deliberately focused on mothers only, as previous research indicated different findings for mothers and fathers (Rodriguez et al., 2019) and sources of stress may differ for mothers versus fathers (Davis & Carter, 2008; Hastings et al., 2005). Our focus was on a specific period, the preschool period (2½–5 years), while children attended an early intervention service, as this may be a particularly important time for parenting stress and child behavior to interact as indicated by previous research (Rodriguez et al., 2019). Drawing from Lin et al.’s (2021) approach, our aim was to investigate longitudinally over an approximate one-year period the relations between internalizing, externalizing, and total behavior that challenges in young children on the autism spectrum and mothers’ parenting stress.

It was hypothesized that higher internalizing and externalizing behaviors in children on the autism spectrum would be associated with increased parenting stress cross-sectionally (as per Barroso et al., 2018; Yorke et al., 2018). Given mixed results, longitudinal hypotheses were tentative. It was predicted that time 1 (T1) internalizing and externalizing behavior in young children on the spectrum may significantly contribute to increased time 2 (T2) parenting stress one year later, after controlling for T1 parenting stress. Further, it was tentatively hypothesized that T1 parenting stress may predict increased T2 internalizing and externalizing behaviors in children on the autism spectrum, after controlling for T1 internalizing and externalizing behaviors. Autism characteristics and age were variables controlled in the analyses.

## Method

### Participants

Participants were 93 mother–child dyads drawn from existing data collected as part of usual service delivery at an Australian autism early learning and care program. Data regarding attrition from the program were not available from the service. All children were attending the early learning and care program between assessment periods (time between, *M* = 11.74 months, *SD* = 2.47). This program is center-based and delivered in an autism-specific group-based early learning context (ages 2½ to 6 years). The program is consistent with a naturalistic developmental behavior intervention model (Schreibman et al., 2015). For a description of the program see Paynter et al. (2012).

Inclusion criteria for the present study were (1) verified autism diagnosis, and (2) maternal completion of a PSI (Abidin, 1995) and completion of the DBC (Einfeld & Tonge, 2002) at intake and 12-months or exit (whichever came first). To enter the service, all children required a formal diagnosis of autism spectrum disorder from a medical practitioner (e.g., pediatrician) or multidisciplinary team using the Diagnostic and Statistical Manual of Mental Disorders—5th edition criteria (DSM-5, American Psychiatric Association, 2013). Existing diagnoses of autism spectrum disorder (DSM-5) were verified for the purpose of this research using the Social Communication Questionnaire (SCQ; Rutter et al., 2003) for most (*n* = 88) participants. Where this was not completed by parents, or a score below the cut-off was obtained, the Autism Diagnostic Observation Schedule-2 (ADOS-2) comparison score (≥ 5 +) was used to verify (*n* = 7, SCQ < 11; *n* = 1, SCQ missing). Participants were an average age of 45.44 months (*SD* = 9.22, range 27.89- 65.84), and included 77 males and 16 females, see Table 1 for further demographics.

**Table 1:** Demographics of participants

	Percentage	Min	Max	M	SD
English as Primary Language	88%				
Married Parents	66%				
Number of Siblings		0	4	1.03	.88
Presence of Co-occurring Conditions	13%				
Time Interval between T1 & T2 Assessments (Months)		5.72	17.25	11.74	2.47
Adaptive Behavior Composite (VABS)		44	98	69.05	9.93
Verbal Developmental Quotient (MSEL)		12.16	109.63	44.44	20.69
Nonverbal Developmental Quotient (MSEL)		24.95	133.34	58.85	19.72

*Note. Min* = minimum; *Max* = maximum; *M* = mean; *SD* = standard deviation. The Adaptive Behavior Composite (standard score) is a global measure of adaptive functioning based on performance on all individual scales from the Vineland Adaptive Behavior Scale (VABS; Sparrow et al., 2005). The verbal and nonverbal developmental quotients are calculated from four subscales of the Mullen Scales of Early Learning (MSEL; Mullen, 1995) as detailed below. Co-occurring conditions included attention deficit hyperactivity disorder, cerebral palsy, chromosomal deletion syndrome, epilepsy, G6PD deficiency, global developmental delay, left congenital torticollis, Kabuki syndrome, Fragile X, and speech difficulties

### Procedure

Data were extracted from an existing database from an Australian center-based early intervention service collected between 2014 and 2017. All parents had provided signed informed consent for their data to be included in this database, the study was covered by ethical approval from the Griffith University Human Research Ethics Committee (Approval number 2014/656), and gatekeeper approval (i.e., consent) from the autism early intervention service was granted. Data were collected at intake to the service, after 12 months, and/or on exit, whichever was first, by the service. Where more than two time points were collected the first two were shared for the study (*M* = 11.74 months, *SD* = 2.47, range 5.72–17.25 months). Data were shared for the purpose of this study for participants with T1 (intake) and T2 (12-months or exit) results on child behavior and parenting stress, and their T1 autism characteristics, cognitive functioning, and adaptive functioning.

### Measures

#### Demographics

Demographics included age at each assessment, diagnosis of child, parental marital status, and primary language spoken at home, see Table 1.

#### Autism Characteristics

The Social Communication Questionnaire: Current Form (SCQ, Rutter et al., 2003) is a 40-item dichotomous measure of potential autism characteristics which yields a total raw score. The SCQ has good psychometric properties including high sensitivity and specificity (Chandler et al., 2007) and convergent validity (Eaves et al., 2006). A cut-off of 11 was used to verify diagnosis based on previous research with preschoolers (Eaves et al., 2006) and as validated as showing maximum sensitivity and specificity in previous research with younger children (aged 17–45 months; Wiggins et al., 2007). The SCQ total score has been used in previous research to verify diagnosis for research including with children of a similar age range (e.g., Paynter et al., 2018b; Westerveld et al., 2020). The total raw score data collected at intake (T1) were used to verify diagnosis.

The Autism Diagnostic Observation Schedule Second Edition (ADOS-2, Lord et al., 2012) is a semi-structured observation which examines social functioning, communication, and repetitive behaviors. It was administered by a staff member employed by the early intervention service who was either a research reliable trained assessor (research and assessment manager who was a registered psychologist) or one of their staff (completed or in-progress bachelor degree in a relevant field such as psychology, education, or speech pathology) who had been trained to reliability with them. All children had completed an ADOS-2 and scores from the administration on intake (T1) to the service were used. It was used to confirm diagnosis using the comparison score where an SCQ was not available or the SCQ score was below the cut-off. The ADOS-2 comparison score was used as a measure of autism characteristics in each of the structural equation models as outlined in data analysis and screening.

#### Adaptive Functioning

The Vineland Adaptive Behavior Scale-2nd Edition (VABS-2, Sparrow et al., 2005) is a parent/carer report which assesses children’s adaptive functioning. It measures communication, daily functioning, socialization, and motor skills, and together scores on these domains are used to calculate an adaptive behavior composite. The adaptive behavior composite standard score was used to describe the sample. The VABS-II has been used widely with children on the spectrum (Yang et al., 2016) and it shows excellent psychometric properties including split-half reliability and test–retest reliability (Sparrow et al., 2005).

#### Verbal and Non-Verbal Functioning

The Mullen Scales of Early Learning (MSEL, Mullen, 1995) is a developmental assessment and includes subscales of receptive and expressive language, visual reception, and fine motor. A fifth gross motor scale (ceiling of three years) was not administered as it was not required to calculate verbal or non-verbal functioning. The manual reports good internal reliability (α = 0.75–0.8; Mullen, 1995). Developmental quotients (dividing the age equivalent by chronological age multiplied by 100) were used for analyses as many children in this population do not attain the minimum score to calculate meaningful standard scores (Paynter et al., 2018b). Consistent with previous research on children on the spectrum (e.g., Paynter et al., 2018b), developmental quotients were calculated for verbal (averaging receptive and expressive language developmental quotients) and non-verbal (averaging fine motor and visual reception developmental quotients) composites to describe the sample, see Table 1.

#### Child Behavior

The Developmental Behavior Checklist (DBC, Einfeld & Tonge, 2002) parent form, is a 96-item questionnaire measure of emotional and behavioral challenges in children and adolescents with intellectual and developmental disabilities. It has five subscales, including anxiety, disruptive/antisocial, communication disturbance, social-relating, and self-absorbed which are combined for a total score. The raw scores for the anxiety subscale were used to measure internalizing behaviors. The raw scores for the disruptive/antisocial scale were used to measure externalizing behaviors. The total scores across all five subscales were used to measure total behaviors that challenge. The DBC has high concurrent validity with other challenging behavior measures (Rice et al., 2018) and high internal consistency, criterion validity, test-retest reliability, and inter-rater reliability (Einfeld & Tonge, 1995). It has been used previously with young children on the autism spectrum (e.g., Adams et al., 2019).

#### Parenting Stress

The Parenting Stress Inventory-Short Form (PSI-SF, Abidin, 1995) measures three domains: difficult child, parental distress, and parent–child dysfunctional interaction. Analyses were conducted separately for both the raw score of the parental distress subscale (PSI-PD) and the raw total score (PSI Total, all three domains) to compare results between different ways of operationalizing parenting stress, given the possible confound between child behavior as both dependent and independent variables when the total score is utilized (as noted by Bohadana et al., 2019; McStay et al., 2014). Previous research has demonstrated excellent internal consistency for the parental distress subscale (e.g., α = 0.86 and 0.90 respectively in Bohadana et al., 2019; McStay et al., 2014) and for the total score (e.g., α = 0.95, Paynter et al., 2018b).

#### Data Analysis and Screening

Descriptive statistics and bivariate correlations between T1 and T2 variables of interest were conducted to describe the initial data and for screening (i.e., to screen for collinearity and to check for data entry errors). Correlations were also conducted controlling for age. Cross-lagged autoregressive models were used to assess the longitudinal relations between parenting stress and child challenging behavior. The autoregressive pathways allow for the growth in the target constructs (i.e., child behaviors that challenge and parenting stress) to be examined, while cross-lagged effects allow for the investigation of (1) the effects of parenting stress levels on later child behaviors that challenge levels, and (2) the effects of child behaviors that challenge levels on later parenting stress levels, while controlling stability effects.

A series of six cross-lagged autoregressive models were developed to explore the inter-relations between parenting stress and child challenging behavior. These models manipulated the measure of child behavior (DBC internalizing/anxiety subscale, DBC externalizing/disruptive behavior subscale, or total score) and parenting stress (PSI-PD or PSI total). For each model, T1 parenting stress and T1 child behavior (DBC internalizing/anxiety subscale, DBC externalizing/disruptive behavior subscale, or total score) were used as direct predictors of both T2 parenting stress and T2 child behavior (DBC internalizing/anxiety subscale, DBC externalizing/disruptive behavior subscale, or total score paired respectively), and therefore included both autoregressive and cross-lagged effects. All manifest variables were regressed on age and ADOS-2 comparison scores (though these regressions are not shown in figures which depict the final parsimonious models). Therefore, any relations between the manifest variables are independent of the shared variance attributable to age and autism characteristics. To obtain the most parsimonious model, any non-significant paths were removed from the initial model iteratively. This did not significantly impact model fit for the final models.

All six models were estimated using MPlus 8.0 (Muthén & Muthén, 1998–2017). Assumption tests indicated that multicollinearity was not a concern for any of the models (all VIFs < 1.38) and all data were normally distributed (Kline, 2010). The resulting models were deemed to be a good fit to the data based on the following fit indices: non-significant chi-square value, comparative fit index (CFI) > 0.95 (> 0.90 is considered acceptable; Brown, 2006), standardized root mean square (SRMR) < 0.08, and root mean square error of approximation (RMSEA) < 0.06 (Hu & Bentler, 1999).

## Results

### Concurrent Associations

Descriptive statistics for parenting stress and child behavior measures are reported in Table 2. Correlations, in which missing data were handled by pairwise deletion, are reported in Table 3. No significant correlations were found between levels of autism characteristics (ADOS-2) and parenting stress (PSI-PD nor PSI total) at either time point, with negative correlations with small effects (< 0.1; Cohen, 1992). However, significant negative correlations of small to medium effect size were observed between autism characteristics (T1) and internalizing (DBC anxiety subscale at T1, *r* = − 0.22; and T2, *r* = − 0.27), externalizing (DBC disruptive behavior subscale at T1, *r* = − 0.26, and T2, *r* = − 0.39), and total behaviors (DBC total score, at T1, *r* = − 0.23; and T2, *r* = − 0.22), see Table 3. At T1 and T2, all child behavior measures positively correlated significantly with total parenting stress (PSI total) at the same time point (i.e., within T1 and within T2) with medium effects (*rs* = 0.30–0.49). Different results for child behavior and parental distress (PSI-PD) were found within each timepoint, see Table 3. At T1, internalizing, externalizing, and total child behavior positively correlated with small to medium effects (*rs* = 0.22–0.31) with parental distress, yet at T2 neither form of child behavior (i.e., internalizing/anxiety and externalizing/disruptive), nor the total DBC score, were significantly correlated in zero order correlations with parental distress.

**Table 2:** Descriptive statistics for parenting stress and child behavior measures at time 1 (T1) and time 2 (T2)

Variable	T1			T2		
	M	SD	Range	M	SD	Range
Autism Characteristics (SCQ total)	18.73	6.02	7–34	–		*–*
Autism Characteristics (ADOS-2 Comparison Score)	5.92	1.97	1–10	*–*		*–*
Internalizing Behavior (DBC anxiety mean score)	.78	.39	.11–1.67	.66	.33	.11–1.44
Externalizing Behavior (DBC disruptive mean score)	.62	.34	0–1.56	.55	.31	.04–1.74
Total Behavior (DBC total score)	61.89	22.57	11–112	54.05	20.37	16–107
Parenting Stress: Parental Distress (PSI-PD)	32.61	8.88	13–60	31.99	9.78	12–54
Parenting Stress: Total (PSI Total Score)	98.00	19.94	54–160	93.58	20.93	46–160

*Note.* T1 = time 1; T1 = time 2; *M* = mean; *SD* = standard deviation; SCQ = Social Communication Questionnaire (Rutter et al., 2003); ADOS-2 = Autism Diagnostic Observation Schedule 2 (Lord et al., 2012); DBC = Developmental Behavior Checklist (Einfeld & Tonge, 2002); PSI = Parenting Stress Index: Short Form (Abidin, 1995)

**Table 3:** Correlations between measures of autism characteristics, behavior and parental stress

	1	2	3	4	5	6	7	8	9	10	11
Time 1											
1. Autism Characteristics: ADOS-2 (comparison score)	–	−.25*	−.24*	−.23*	−.06	−.10	−.28**	−.38**	−.21*	−.08	−.15
2. Internalizing Behaviors: DBC Anxiety	−.22*	−	.66**	.75**	.23*	.44**	.66**	.41**	.35**	.16	.19
3. Externalizing Behaviors: DBC Disruptive	−.26*	.63**	−	.87**	.30**	.49**	.52**	.58**	.39**	.15*	.27*
4. Total Behaviors: DBC Total	−.23*	.74**	.86**	−	.29**	.29**	.54**	.51**	.51**	.24*	.25*
5. Parental Distress: PSI-PD	−.06	.22*	.31**	.29**	−	.83**	.11	.15	.06	.65**	.49**
6. Parental Total Stress: PSI-Total	−.11	.42**	.49**	.49**	.83**	−	.35**	.31**	.26*	.61**	.60**
Time 2											
7. Internalizing Behaviors: DBC Anxiety	−.27**	.66**	.51**	.54**	.11	.35**	−	.67**	.69**	.12	.30**
8. Externalizing Behaviors: DBC Disruptive	−.39**	.38**	.58**	.50**	.16	.31**	.66**	−	.83**	.22*	.39*
9. Total Behaviors: DBC Total	−.22*	.34**	.40**	.51**	.06	.26*	.69**	.83**	−	.16	.35**
10. Parental Distress: PSI-PD	−.07	.17	.24**	.24*	.64**	.60**	.12	.20	.15	−	.82**
11. Total Stress: PSI Total	−.14	.19	.26**	.25*	.49**	.59**	.30**	.38**	.35**	.82**	−
12. Time 1 age (months)	−.17	−.14	.12	.01	.05	.05	−.01	.16	.08	−.07	−.04

*Note.* Zero order correlations are below the diagonal; partial correlations controlling for age are above the diagonal ** *p* < .01; * *p* < .05. ADOS = Autism Diagnostic Observation Schedule-2 (Lord et al., 2012); DBC = Developmental Behavior Checklist (Einfeld & Tonge, 2002); PSI = Parenting Stress Index Short Form (Abidin, 1995)

### Cross-Lagged Autoregressive Models

#### Parental Distress and Child Behavior

**Fig. 1:**
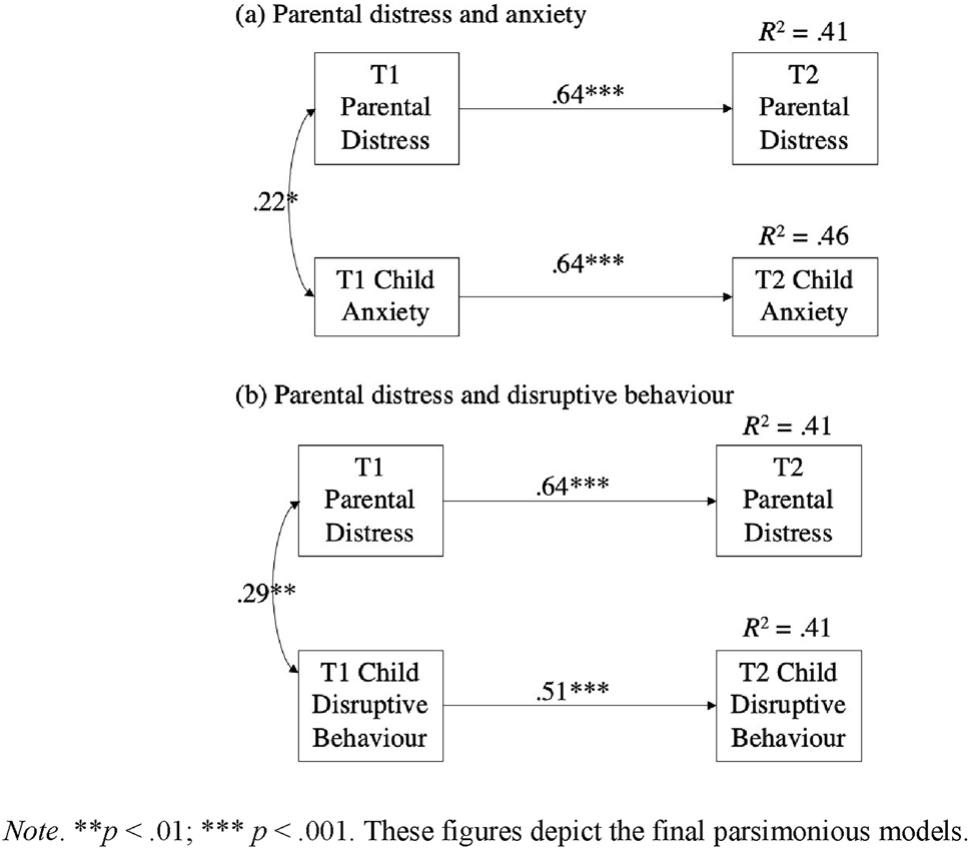
Manifest cross-lagged autoregressive model predicting parental distress and (**a**) child anxiety or (**b**) disruptive behavior

The first two models examined the relations between individual subscales of the DBC (i.e., internalizing/anxiety and externalizing/disruptive behavior) and parental distress (PSI-PD), see Fig. 1. The removal of the non-significant pathways, including the cross-lagged effects, did not result in any appreciable loss in fit for either the internalizing/anxiety (χ^2^ difference (2) = 0.37, *p* = 0.83) or externalizing/disruptive behavior (χ^2^ difference (2) = 0.42, *p* = 0.81) models. The final parsimonious models provided a very good fit to the data (anxiety: χ^2^(3) = 0.47, *p* = 0.92, RMSEA = 0.00 (90% CI = 0.00–0.06), CFI = 1.00, SRMR = 0.01; disruptive behavior: χ^2^ (3) = 1.96, *p* = 0.58, RMSEA = 0.00 (90% CI = 0.00–0.15), CFI = 1.00, SRMR = 0.02), with both models revealing significant stability effects for parent distress at T1 and T2, and child behavior at T1 and T2. It is worth noting that, although not reported in the model, autism characteristics did not significantly predict parental distress at T2 in either the anxiety (standardized path coefficient = − 0.04, *p* = 0.61) or disruptive behavior model (standardized path coefficient = − 0.04, *p* = 0.61).

#### Parental Stress Total and Child Behavior

**Fig. 2:**
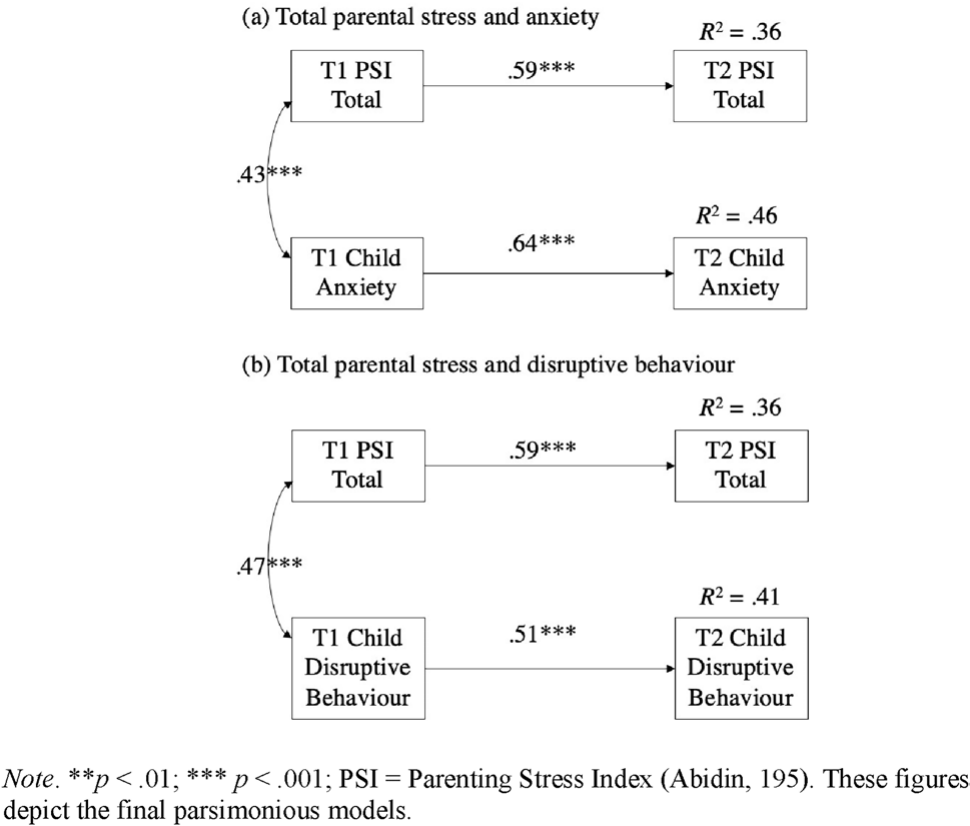
Manifest cross-lagged autoregressive model predicting total parental stress score and (**a**) child anxiety or (**b**) disruptive behavior

The second two models explored the autoregressive and cross-lagged relations between child behavior (i.e., DBC internalizing/anxiety and externalizing/disruptive behavior) and total parental stress score (PSI total) at T1 and T2 (See Fig. 2). The removal of non-significant pathways did not impact model fit (anxiety: χ2 difference (2) = 2.51, *p* = 0.28; disruptive behavior: χ2 difference (2) = 0.54, *p* = 0.76), therefore the parsimonious models were accepted. The final model for anxiety (Fig. 2a) provided a good fit to the data, χ2 (3) = 6.29, *p* = 0.10, RMSEA = 0.11 (90% CI = 0.00–0.23), CFI = 0.97, SRMR = 0.03, demonstrating large significant stability effects between PSI total scores at T1 and T2, and significant stability effects between anxiety at T1 and T2. Much like the internalizing/anxiety path model in Fig. 1a, autism characteristics were not significant predictors of parental stress at T2 (standardized path coefficient = − 0.09).

For externalizing/disruptive behavior, see Fig. 2b, after the removal of non-significant paths, including the cross-lagged effects, the path model provided an acceptable fit for the data when considering CFI and SRMR, χ2 (3) = 9.85, *p* = 0.02, RMSEA = 0.16 (90% CI = 0.06–0.27), CFI = 0.94, SRMR = 0.04. It is clear this model has a poorer fit than the corresponding externalizing/disruptive behavior model presented in Fig. 1b. Autism characteristics were not considered a significant predictor of parenting stress at T2 (standardized coefficient estimate = − 0.09).

#### Parenting Stress (Total Score and Parental Distress) and Total Child Behavior

**Fig. 3:**
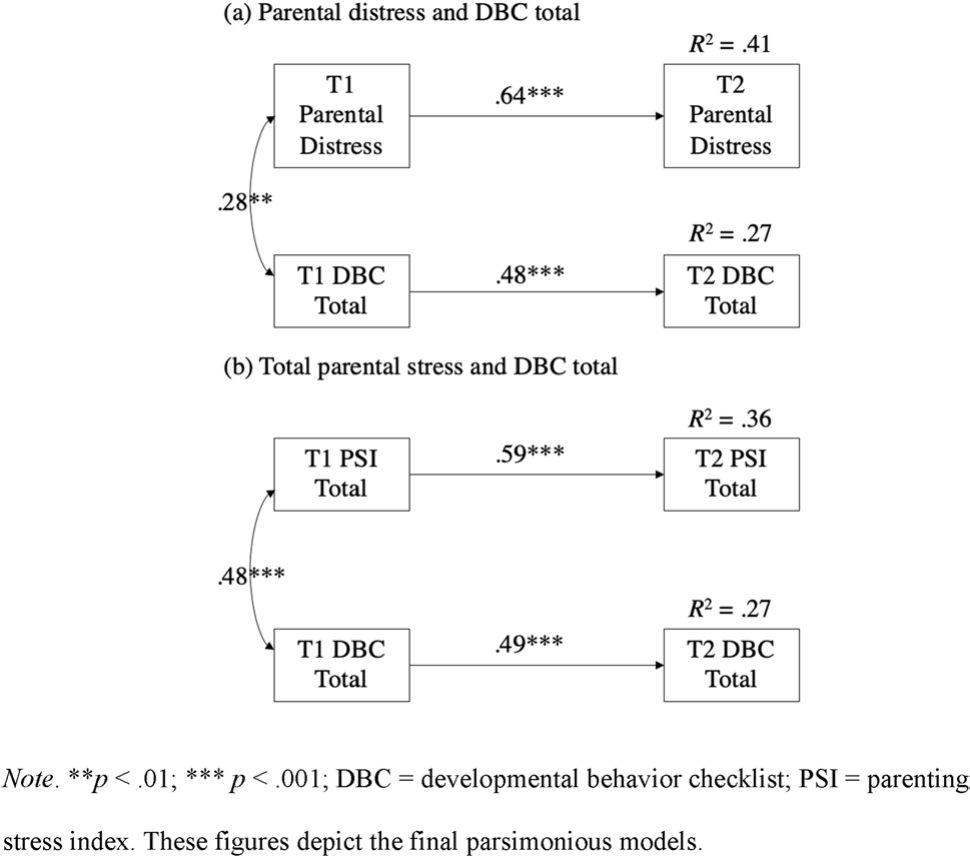
Manifest cross-lagged autoregressive model predicting total behaviors that challenge score and (**a**) parental distress or (**b**) total parental stress score

The final two models examined the relations between either parental stress as measured using the PSI-PD subscale alone or the PSI total score and total child behavior. Therefore, behavior in these models included both internalizing (DBC anxiety) and externalizing (DBC disruptive behavior) behaviors. As with the previous models, only the paths representing the autoregressive effects were significant, while all cross-lagged effects were non-significant, and were therefore removed from the model (*p* > 0.05). The iterative removal of the non-significant paths did not cause any appreciable loss of fit for either the PSI-PD model (see Fig. 3a; χ2 difference (2) = 1.85, *p* = 0.40) nor the PSI total score model (see Fig. 3b; χ2 difference (2) = 0.74. *p* = 0.69).

The final, parsimonious model examining the relations between child behavior and parental distress alone provided a very good fit to the data, χ2 (3) = 3.08, *p* = 0.38, RMSEA = 0.02 (90% CI = 0.00–0.18), CFI = 0.999, SRMR = 0.02. In contrast, the model incorporating the total parental stress scale (instead of the PSI-PD subscale) had a poorer fit to the data, χ2 (3) = 9.16, *p* = 0.03, RMSEA = 0.15 (90% CI = 0.05–0.27), CFI = 0.94, SRMR = 0.04, although CFI and SRMR were still within the acceptable range. Both models demonstrated large stability estimates between the corresponding T1 and T2 variables. Again, either when entered as the total parental stress score (standardized coefficient estimate = − 0.09) or the PSI-PD subscale (standardized coefficient estimate = − 0.04), autism characteristics were not significantly related to parental stress.

## Discussion

Our aim was to investigate the relations between behaviors that challenge in young children on the autism spectrum and their mothers’ parenting stress. As predicted, the total parent stress score showed significant relations with all measures of child behavior cross-sectionally. However, the more specific measure of parental distress, did not show significant relations at T2. Parenting stress and child behavior measures showed stability when measured approximately one year later. In contrast to expectations, we found no cross-lagged significant pathways within our models that controlled for stability effects (i.e., stability of T1 to T2 parenting stress and child behaviors that challenge). Specifically, in our models neither measure of parent stress (PSI-PD nor PSI total) at T1 predicted any measure of child behavior at T2 and no measure of child behavior (internalizing, externalizing, nor total) at T1 predicted either measure of parenting stress at T2. We discuss these key findings, limitations, future direction, and implications below.

We did not find autism characteristics were significantly associated with concurrent parenting stress, consistent with Lin et al. (2021). This suggests a child's level of autism characteristics may not lead to higher stress for their parents. Instead, higher stress may be due to factors associated with caregiving for an autistic child such as increased financial outgoings (e.g., costs of accessing additional supports), associated behaviors that challenge (at least cross-sectionally), or how parents cope with caregiving (e.g., Paynter et al., 2013). Further, in contrast to some of the previous research (Bader et al., 2015; Falk et al., 2014), the level of autism characteristics did not influence the association (i.e., did not significantly impact the model fit) between parenting stress and child behaviors over time. This suggests other variables should be considered in understanding the trajectory of parenting stress and child behaviors over time as discussed further below.

Our finding of a negative relationship between autism characteristics and behavior contrast with Lin et al. (2021). It may be that children with lower autism characteristics have greater insight into their challenges or differences which leads to expressing these challenges more frequently or at a higher intensity in their behavior. Alternatively, differing findings may reflect differences in assessment measures used between studies. We used the DBC to measure child behaviors which may be more sensitive to autism-relevant behaviors than the CBCL as used by Lin et al. given that the CBCL was not designed for this population. Mixed findings suggest future research is needed to evaluate if the measurement used impacts associations between autism characteristics and behavior.

Higher total parenting stress was significantly associated with higher (i.e., amount or intensity) levels of child behaviors that challenge in cross-sectional correlations, which is consistent with previous research (Yorke et al., 2018). Given the correlational nature of this analysis, it is not possible to determine causality. It may be that higher parenting stress impacts on parenting practices (e.g., giving in) that inadvertently reinforce behaviors that challenge. Conversely, observing a child’s higher levels of behaviors that challenge may be distressing for the parent, and lead to higher parenting stress. This association may be higher for parenting stress measures (i.e., the PSI difficult child subscale and total score which includes this subscale) that include items about the child’s behavior (e.g., about perceptions of a child’s mood or behavior) as opposed to items about parenting stress more generally (e.g., about the parent’s mood or behaviors). As such, the observed relation between the total parent stress score and child behavior may be driven by the parent stress measure and child behavior measures both including items that consider child behaviors. Consistent with this possibility, when we used the parental distress subscale alone (therefore removing the influence or overlap with child behavior apparent in the total score), a significant concurrent correlation between parental distress and child behavior was only found at T1. This is consistent with previous findings that similarly found lower relations with more specific measures of parenting stress such as the parental distress scale (that reflect on parent mood or behaviors only) and higher relations with broader parenting stress measures that included child behavior items (Bohadana et al., 2019; McStay et al., 2014; Yorke et al., 2018). This means in research and practice it is important to select measures that are distinct (e.g., using the PSI-PD subscale rather than total) to avoid overestimating relations, or capturing for example child behavior rather than parenting stress in measurement. Utilizing distinct measures would enable more accurate identification of specific child behavior and/or parenting stress strengths and challenges for targeted support and monitoring of treatment progress.

We found strong stability shown through large effect sizes, of measures of parenting stress and child behaviors at T1 predicting scores at T2, consistent with previous research (Lin et al., 2021; Rodriguez et al., 2019). It may be that parenting stress and/or child behaviors are stable over time and/or are less likely to change over an approximately one-year period. Parenting stress may be stable as perhaps the demands arising from caring for an autistic child in a predominantly neurotypical society such as facing stigma and judgement from others (e.g., Rusu et al., 2024) may be ongoing, and difficult to change at a societal level, thus maintaining parent reported stress. Stability in child behavior may be explained by a focus in the center-based intervention program on children’s behavior and skills at the center, with a lack of generalization of skills to contexts observed by parents (i.e., for ratings). This may indicate a need for broader supports (e.g., training for parents in how to support or respond to children’s behavior that challenges) in the home setting. This is consistent with the broader literature that has found supports for autistic children show greater effects on outcomes measured at proximal (e.g., behavior with staff at the center) than distal (e.g., behavior at home with parents) levels (see systematic review by Sandbank et al., 2020).

Stability effects observed between measures repeated at T1 and T2 may also reflect the timing of assessments at two potentially challenging times for parents and their children. At the intake assessment, families may have just learned about their child’s autism diagnosis, and thus may have been overwhelmed with navigating this information. At the second assessment, approximately 12 months later which was the time of leaving the service for many families, they may have been faced with navigating transitioning from the early learning and care service and into formal education. For parents, this may have led to high levels of parenting stress at each time point. For children, both time points may have coincided with times of transitioning to new routines and environments (e.g., visiting potential schools, see Fontil et al., 2020) which could lead to high levels of behaviors that challenge at each time point. More targeted or frequent assessments of child behavior and parenting stress, including use of multiple informants across settings, may be useful to monitor changes in response to specific stressors, in response to targeted supports, and throughout receipt of early learning and care services and specific programming.

We did not find evidence of bidirectional effects of parenting stress and child behavior in our models controlling for stability effects, consistent with Peters-Scheffer et al. (2012). That is, parenting stress did not significantly predict later child behavior, nor the reverse. Similarly, Lin et al. (2021), also found non-significant effects between a specific measure of parental distress and later child behavior. Thus, clinicians should not assume that a child’s behavior is causing a parent’s later stress, nor that parenting stress will directly lead to later child behaviors that challenge. This highlights the importance of directly measuring each construct and providing targeted supports for each. It may be that while parenting stress and child behavior are associated concurrently, but over time other factors may impact relations. For example, the parenting practices that parents employ to support or respond to children’s behaviors may be impacted by stress. These parenting practices could increase (e.g., controlling parenting practices such as harsh punishment) or decrease (e.g., mindful parenting, i.e., paying attention to parenting non-judgmentally) child behavior (see systematic review, Suvarna et al., 2024). Additionally, avoidant coping strategies (e.g., using alcohol or drugs as a strategy to cope with responding to children’s behavior) may increase parenting stress (Paynter et al., 2013; Stuart & McGrew, 2009). Further, the interpretation or meaning assigned to their child’s behavior such as forming negative appraisals can negatively impact parenting stress (Paynter al., 2013). External supports such as social support, may mitigate parental stress in response to child behavior (Boyd, 2002). Coping strategies, appraisals, and supports are captured in theoretical models of adaptation previously applied to autism (such as the Double ABCX Model as used in Paynter et al., 2013) and may be useful to include in future supports for decreasing levels of parenting stress.

Three of our key findings include stability effects between like measures at T1 and T2, a lack of relation between autism characteristics and parenting stress, and that earlier parenting stress did not predict later child behaviors. Stability effects and lack of relation between autism characteristics and parenting stress are consistent with Lin et al. (2021). In contrast, Lin et al. found higher levels of earlier parenting stress predicted higher levels of later child externalizing behaviors. Cultural differences may explain differences between our study conducted in Australia and Lin et al.’s (2021) findings from Taiwan. That is, there may be different expectations or beliefs regarding autism, behavior, and/or parenting between cultures which may relate to differences in levels of stress, strategies parents use to cope, or supports available (Lin et al., 2011). For example, lower knowledge of autism and greater stigma around autism have been reported in studies in Asia compared to the United States (Yu et al., 2020). Further, differences in coping strategies of mothers of autistic children cross-culturally have also been found. Taiwanese mothers were reported to use more problem-focused and emotion-focused coping styles than mothers in the United States (Lin et al., 2011).

Our sample of children were older compared to children in other studies investigating relations between parenting stress and child behavior over time (Lin et al., 2021). Longitudinal relations between parenting stress and child behavior may vary with age as indicated in previous research (Rodriguez et al., 2019). Specifically, child age and access to early intervention may have impacted findings across studies. Younger children, as in Lin’s study (*M* = 25.68 months), may have greater contact time with parents as they did not appear to be enrolled in full-time center-based intervention with Lin reporting most (71%) received occupational therapy or speech therapy (63%) services only. In contrast, our sample were older (*M* = 45.44 months), and all were receiving center-based early intervention services. There may be greater opportunity for transactional effects (i.e., parenting stress impacting child behaviors that challenge or child behaviors that challenge impacting parenting stress) in younger age groups due to parents and their child spending more time together at a younger age. We hypothesize, there may also be other changes in the parent–child dynamic as children age, such as parental adjustment to diagnosis, changes in child characteristics (e.g., development of skills or regression may positively or negatively impact stress respectively), increases in parenting skills or increases in coping abilities. Further, we hypothesize that the center-based program may have provided a form of respite to families, so there may have been less opportunity for parenting stress to impact on child behaviors explaining the non-significant effects over time.

### Limitations and Future Directions

Our research importantly addressed previous research limitations (i.e., controlled for stability effects, included autism characteristics, used a measure of behavior designed for developmental disabilities, and compared specific vs. broad measures of parenting stress); however, we acknowledge the following limitations. First, we investigated mothers only and there is a need for further research with fathers given sources of stress may differ (Davis & Carter, 2008; Hastings et al., 2005). Future research with fathers would be of value to inform targeted supports for each parent/caregiver (see Paynter et al., 2018a). Second, data were not collected on broader caregiver characteristics (e.g., parental mental health, parental autism characteristics, parenting practices, or coping strategies). These additional measures may provide further insights into the interaction between child behavior and parenting stress over time by identifying risk and protective factors, as well as factors potentially amenable to change. Future longitudinal research that draws from theoretical models of adaptation such as the Double ABCX model (McCubbin & Patterson, 1983), as used in cross-sectional research (McStay et al., 2014; Paynter et al., 2013), would be of value in identifying potential risk and protective factors to explore. This research could include variables from these models such as social support, coping strategies, or appraisals to delineate protective and risk factors, and mediators of outcomes over time. This could inform and extend existing parent support research into cognitive behavioral and psychoeducational interventions (see review, Bourke-Taylor et al., 2021).

Finally, we acknowledge potential sampling bias through using data drawn from one specific early intervention service in one Australian state. The content of the specific program may have impacted results, and attrition data were not available. Further, the length of follow-up varied from T1 to T2 between individual participants from 5.72 to 17.25 months and may have impacted results. Future research should include tracking of attrition and whether those who do not complete all assessment measures over time differ from those who do complete all measures, and whether length of time between measures impacts relations. In addition, while access to this service could be partially funded through government financial supports, families contributed financially to their children’s programs potentially biasing towards higher socio-economic status (SES) participants. Parents from higher SES backgrounds may have access to greater resources to support children’s outcomes (such as affording early intervention) while families from lower SES backgrounds may, by necessity, need to prioritize meeting basic needs as per the *Family Investment Model* (Conger & Donnellan, 2007). SES could subsequently impact on the associations observed between child behavior and parenting stress. Unfortunately, further information on families (e.g., SES) were not available to analyze this possibility and may be of value in future research. Future research investigating relations between child behaviors and parenting stress receiving a range of services would also be of value in exploring other potentially important mediators.

### Conclusion

These findings highlight the need to assess rather than to assume that child behaviors that challenge *will* have a strong direct effect on later parenting stress for mothers over time or vice versa, and to question this potential assumption that may be drawn from cross-sectional findings. Our findings highlight the need for analyses to control for stability of parenting stress and challenging behavior in longitudinal research. We also emphasize the need to consider broader psychological, social, and contextual factors that may impact parenting stress and outcomes for children and both parents. Of concern, is the relative stability of parenting stress and child behaviors which may impact on quality of life for children and their mothers and highlights the need for specific supports to improve outcomes.

## Footnotes

1 The terms “on the autism spectrum” and “on the spectrum” in latter occurrences, are used in line with the preferences of the autistic and autism community (Bury et al., 2020; Kenny et al., 2016).
